# Genomic and transcriptomic-based analysis of agronomic traits in sugar beet (*Beta vulgaris* L.) pure line IMA1

**DOI:** 10.3389/fpls.2022.1028885

**Published:** 2022-10-13

**Authors:** Xiaodong Li, Wenjin He, Jingping Fang, Yahui Liang, Huizhong Zhang, Duo Chen, Xingrong Wu, Ziqiang Zhang, Liang Wang, Pingan Han, Bizhou Zhang, Ting Xue, Wenzhe Zheng, Jiangfeng He, Chen Bai

**Affiliations:** ^1^ Inner Mongolia Academy of Agricultural and Animal Husbandry Sciences, Hohhot, China; ^2^ Life Science College of Fujian Normal University, Fuzhou, China; ^3^ Inner Mongolia Key Laboratory of Sugarbeet Genetics & Germplasm Enhancement, Inner Mongolia Academy of Agricultural and Animal Husbandry Sciences, Hohhot, China

**Keywords:** *Beta vulgaris*, whole-gene sequencing, whole genome duplications (WGD), gene family, genome wide association study (GWAS), male sterility

## Abstract

Sugar beet (*Beta vulgaris* L.) is an important sugar-producing and energy crop worldwide. The sugar beet pure line IMA1 independently bred by Chinese scientists is a standard diploid parent material that is widely used in hybrid-breeding programs. In this study, a high-quality, chromosome-level genome assembly for IMA1was conducted, and 99.1% of genome sequences were assigned to nine chromosomes. A total of 35,003 protein-coding genes were annotated, with 91.56% functionally annotated by public databases. Compared with previously released sugar beet assemblies, the new genome was larger with at least 1.6 times larger N50 size, thereby substantially improving the completeness and continuity of the sugar beet genome. A Genome-Wide Association Studies analysis identified 10 disease-resistance genes associated with three important beet diseases and five genes associated with sugar yield per hectare, which could be key targets to improve sugar productivity. Nine highly expressed genes associated with pollen fertility of sugar beet were also identified. The results of this study provide valuable information to identify and dissect functional genes affecting sugar beet agronomic traits, which can increase sugar beet production and help screen for excellent sugar beet breeding materials. In addition, information is provided that can precisely incorporate biotechnology tools into breeding efforts.

## Introduction

Sugar beet (*Beta vulgaris* L.) is in the Caryophyllales in the family Chenopodiaceae. The chromosome number of cultivated sugar beet is2n =2x= 18, with a predicted genome size of714 to 758 Mb (Arumuganathan and Earle, 1991). Sugar beet is an important biennial root crop cultivated in temperate climate regions with outstanding sugar-producing capability. Sugar beet was originated by selecting lines with high sugar content in the storage root from hybridizations between typical fodder beet and chardin the late eighteenth century (Eberhard, 1989) and thus is one of the most recently domesticated crops.

Sugar beet productivity is threatened by various pathogens, including bacteria, fungi, viruses, and nematodes (Larson et al., 2006; Saleh et al., 2011; Strausbaugh and Eujayl, 2018). Molecular breeding approaches have been used to create resistant or high taproot-yield sugar beet germplasms to increase production while greatly decreasing time, effort, and costs (Boyd et al., 2013). Many genes associated with important agronomic traits have been identified in sugar beet, including those responsible for nematode resistance (Cai et al., 1997), life cycle adaptation (Pin et al., 2012), cytoplasmic male sterility (Matsuhira et al., 2012), bolting tolerance (Hébrard et al., 2016), and salt tolerance (Sahashi et al., 2019). A wide range of sequence-based genetic and genomic resources are emerging for sugar beet. Single Nucleotide Polymorphism based genetic and physical maps have been constructed (Dohm et al., 2012; Holtgräwe et al., 2014), and transcriptome profiles have been analyzed to reveal important metabolic pathways and stress-responsive genes (Mutasa-Göttgens et al., 2012; Lv et al., 2018; Geng et al., 2019; Zou et al., 2020). Several sugar beet genomes have been assembled, including chromosome-level assemblies of double-haploid line RefBeet (Dohm et al., 2014) and the five-generation inbred line EL10 (Funk et al., 2018). Genome-wide identification and characterization of various important functional genes have also been reported (Stracke et al., 2014; Funk et al., 2018; Wang et al., 2019; Wu et al., 2019a; Wu et al., 2019b).

However, insufficient publicly available genetic resources and innovative germplasms are two major factors that limit the development of superior sugar beet cultivars. In this study, the chromosome-level genome assembly of the first Chinese native sugar beet line IMA1 was built by combining IlluminaHiseq, PacBio SEQUEL, and Hi-C sequencing platforms. Compared with previously released sugar beet assemblies, the new genome was 220 Mblarger with N50 size that was at least 1.6 times larger, thereby greatly improving the completeness and continuity of the sugar beet genome. Seven important beet agronomic traits and disease-resistance characteristics were also assessed by resequencing 114 accessions. In addition, a group of candidate genes associated with male sterility in sugar beet were selected based on q-PCR and transcriptome sequencing.

In conclusion, sequencing, assembly, and annotation of the sugar beet IMA1line provide the foundation for future comparative genomics efforts and phylogenetic reconstructions in the Caryophyllales and eudicots. Furthermore, valuable information is provided to identify and dissect functional genes affecting agronomic traits, which can be used to create breeding materials and to precisely incorporate biotechnology tools into breeding efforts.

## Materials and methods

### Sample collection and processing

*Beta vulgaris* IMA1, an inbreeding line with low level of heterozygosity, was selected for sequencing. Scientists from the Inner Mongolia Academy of Agricultural and Animal Science (IMAAAHS, Hohhot, Inner Mongolia, China) independently developed line IMA1. The line is standard diploid parent material with good combining ability that is widely used in creating sugar beet parent materials and hybrid breeding.

Seeds of IMA1 were planted in one gallon flowerpots filled with organic loamon August 16, 2018, and placed in a greenhouse at IMAAAHS. Greenhouse temperatures were 26°C(day) and 21°C(night). Two months after planting, tender, young, healthy leaf samples were collected and immediately flash-frozen in liquid nitrogen for one hour and then stored at −80°C until DNA and RNA extraction. Voucher specimens of IMA1 were deposited at IMAAAHS with collection number 14.S4006C.

### Sampling germplasms of 114 sugar beet accessions

Test materials were 114 accessions randomly selected from the sugar beet gene bank stored at the Special Crop Research Institute of IMAAAHS. All test materials were planted in the experimental field of IMAAAHS (longitude 40°46′19.43″N, latitude 111°39′44.96″E)in Hohhot, Inner Mongolia, China. The complete data set contained three years (2017 to 2019) of agronomic traits collected in the field. Sugar beets were planted at the beginning of May and harvested at the beginning of October. Each plot was 6 m in length and 55-cm in width. The 114 sugar beet accessions were randomly sampled during the lush growth period. Newly emerged leaves were removed, put into zip lock bags, quickly frozen in a sample box with liquid nitrogen, and placed in a freezer at −80°C.

### Selection of beet accessions for transcriptome analysis

Two pairs of beet lines with differences in male fertility were selected for transcriptome analysis: two male-sterile beet lines MS137 and MS301 and two beet maintainer lines OT152 and OT302. Beet roots that had undergone vernalization were planted in a test field arranged for beet breeding and isolation. On June 20, during the sugar beet budding stage, beet inflorescences with unopened, mature flower buds were selected and snap-frozen in liquid nitrogen.

### DNA sequencing

To extract DNA and total RNA from young and healthy sugar beet leaf tissues, a DNeasy Plant Mini Kit (Qiagen, Germany) and an RNAprep pure Plant Kit (Tiangen, Beijing, China) were used, respectively. The DNA-seq was used to assist genome assembly, and the RNA-seq was used for gene model prediction. Low-quality reads and adaptor sequences were filtered out with the HTQC utility (Yang et al., 2013).

To obtain long reads for genome assembly, long read libraries were constructed using the extracted high-quality DNA in PacBio sequencing. Five SMRT (Single-Molecule Real Time Sequencing) cells were sequenced, and roughly 65.67 Gb of data were generated on a PacBio SEQUEL platform (Menlo Park, CA, USA) (**Supplementary Table 1**). With a genome size of 700 Mb assumed for sugar beet, the sequencing result theoretically represented 94-fold coverage. The average subread length was 10,727 bp, and the N50 length was 17,047 bp. The PacBio sequencing was combined with Illumina sequencing to generate longer scaffold genome assemblies.

### Scaffold-level genome assembly of *Beta vulgaris* IMA1

High-quality Illumina sequences with a *K*-mer size of 17 were counted using the JELLYFISH program (Marçais and Kingsford, 2011). The PacBio sequencing subreads were assembled using Canu v1.7 (Koren et al., 2017). There were two steps of genome assembly polishing to correct random sequencing errors. Aquiver algorithm (Chin et al., 2013) was used to polish the Canu assembly using 50× long PacBio subreads. Next Generation Sequencing (NGS) short reads deliver a read accuracy of over 99% (Dohm et al., 2008). By contrast, with PacBio long reads, the error rate isas high as 15% to 20% (Ono et al., 2013; Ross et al., 2013). Therefore, two rounds of polishing were conducted with 67.22 Gb of Illumina short reads recruited with Pilonv 1.21 (Altschul et al., 1990; Walker et al., 2014). Organellar contigs were also removed by BLAST searches against organellar genomes of sugar beet (chloroplast genome: accession number KR230391.1;mitochondria genome: accession number BA000024.1).

### High-throughput chromatin conformation capture library construction and chromosome assembly

In the current study, the Hi-C approach was used for chromosome-level assembly of sugar beet (Zhang et al., 2018; Chen et al., 2019; Zhang et al., 2020). To construct a Hi-C library, young leaves were cross-linked with formaldehyde and digested with *Dpn*II restriction enzyme overnight. Chimeric junctions were formed followed by biotinylating and proximity ligating sticky ends and then sheared and enriched for fragment sizes from 500 to 700 bp. Chimeric fragments were subjected to PE sequencing on an Illumina HiSeq X ten system (San Diego, CA, United States) with the PE 150 nt mode.

After mapping the clean sequencing reads against the polished sugar beet genome with Bowtie2 software (Langmead and Salzberg, 2012), over 369.4 million PE reads matched unique genomic locations, which were assessed and filtered by the hiclib Python library (Imakaev et al., 2012) and HiC-Pro program (Servant et al., 2015). Mis-joined contigs were corrected with the 3D-DNA pipeline (Dudchenko et al., 2017), and Hi-C-corrected contigs were grouped into pseudo-chromosomes by the ALLHIC pipeline (Zhang et al., 2019) on the basis of relations among valid reads.

### Genome annotation

With gene model parameter strained from *Arabidopsis thaliana*, ab initio predictions were conducted using AUGUSTUS (Stanke and Morgenstern, 2005). Previously published sugar beet genome RefBeet-1.2.2 of sugar beet line RefBeet (Dohm et al., 2014) with accession number GCA_000511025.2 was selected as the reference genome to perform homology annotation. The protein sequences of the RefBeet genome were aligned with those of the new genome by TBLASTN software (Winsor et al., 2016). Gene structures were further predicted by GeneWise (Birney and Durbin, 2000) on the basis of TBLASTN results. The RNA-seq data sampled from leaf tissues were used for Trinity (Haas et al., 2013) *de novo* assembly. Transcript abundance was calculated with RNA-Seq by Expectation-Maximization (RSEM) (Li and Dewey, 2011), and transcripts with Fragments Per Kilobase Million (FPKM) <1 and iso-percentage <3% were filtered out. The PASA program (Haas et al., 2003) was used to construct comprehensive transcripts using the filtered transcripts. Sugar beet transcripts were compared with the UniProt to identify candidates covering ≥95% of any target protein. Homology-based annotation, ab initio, and transcriptome-based gene prediction were combined to generate a protein-coding gene set by using the Evidence Modeler pipeline (Haas et al., 2008).Tandem Repeats Finder (Benson, 1999) and LTR_FINDER (Xu and Wang, 2007) were used to predict repeat elements. Subsequently, assembled genome sequences were aligned to the Repbase TE database (Bao et al., 2015) using Repeat Masker (Tarailo-Graovac and Chen, 2004) to search for sequences of repeat elements. The tRNAscan-SE (Schattner et al., 2005) and rRNAmmer (Lagesen et al., 2007) were used to detect reliable transfer RNA(tRNA) and ribosomal RNA(rRNA) positions, respectively. The small RNAs (sRNAs), microRNAs (miRNAs), and small nuclear RNAs(snRNAs)were predicted by searching the RFAM databases (Gardner et al., 2009) using INFERNAL software (Nawrocki et al., 2009) with the default parameters. For functional annotations, sequence-similarity searches were performed using Blast with *E-*value of 10^−5^ in available protein databases [(Non-Redundant Proteins (NR), Swiss-Prot, Clusters of Orthologous Groups (COGs), Kyoto Encyclopedia of Genes and Genomes(KEGG), and Gene Ontology(GO)].

### Phylogenetic analysis and divergence time estimation

Phylogenetic analysis was conducted using the protein-coding genes of IMA1 and 25 other species. Protein sequence alignments and phylogenetic tree construction were conducted using OrthoFinder software (Emms and Kelly, 2019). Reconstruction of phylogenetic trees was inferred by maximum likelihood (ML), and the estimated divergence time of plant species based on the TimeTree database (Puttick, 2019) (http://www.time.org/) was used to recalibrate the divergence time for the 26 plant species. To identify the expansion and contraction of gene families, CAFE was used (Lu et al., 2017).

### Synteny analysis and whole-genome duplication

The paralogous genes of IMA1 were identified in a BLASTP search (*E*-value cutoff of 1E−5). Synteny and collinearity blocks of those genes were analyzed using MCScanX (Wang et al., 2012). Gene synteny, gene density, and GC content on individual pseudo-chromosomes were mapped by using Circos software (http://www.circos.ca). The synonymous substitution rate (Ks) was calculated using KaKs_Calculatorand the Nei–Gojobori method (Wei and Zhang, 2014).

### Single nucleotide polymorphisms and insertion and deletion calling

Trimmed reads were mapped to the new genome using BWA-MEM (Li, 2013). Average mapping rates were 99.33%, and average genome coverage was 7.72-fold of the reference genome. Mapping results were sorted and duplicate reads marked based on Sambamba (Tarasov et al., 2015). SNPs and InDels of the 114 accessions were called by GATK HaplotypeCaller (Hasanl et al., 2015). The results were calculated using the following parameters: QD < 2.0; MQ < 40.0; FS > 60.0; QUAL <30.0; MQrankSum <−12.5; Read PosRankSum <−8.0 -clusterSize 2 –cluster Window Size 5. The identified SNPs were filtered. High-quality SNPs were defined as only those with a minor allele frequency >0.05 and missing data rate<0.8. SNPs were annotated based on the genome with snpEff (Cingolani et al., 2012). Furthermore, SNPs were classified as coding synonymous SNPs and non-synonymous SNPs, and InDels in exons were grouped based on whether they led to a frameshift.

### Genome wide association study analysis

Genome wide association study was performed by using FaST-LMM (v2.07.20140723) or EMMAX (Kang et al., 2010). A total of3,738,500 SNPs with a minor allele frequency of 0.05 or greater and a missing data rate of 80% or less in the entire population were used for GWAS. A Bonferroni correction was used to determine the genome-wide significance thresholds of the GWAS, based on a nominal level of −log10(P) values of 5.

## Results

### Sequencing and assembly of IMA1 genome

**Figure 1:**
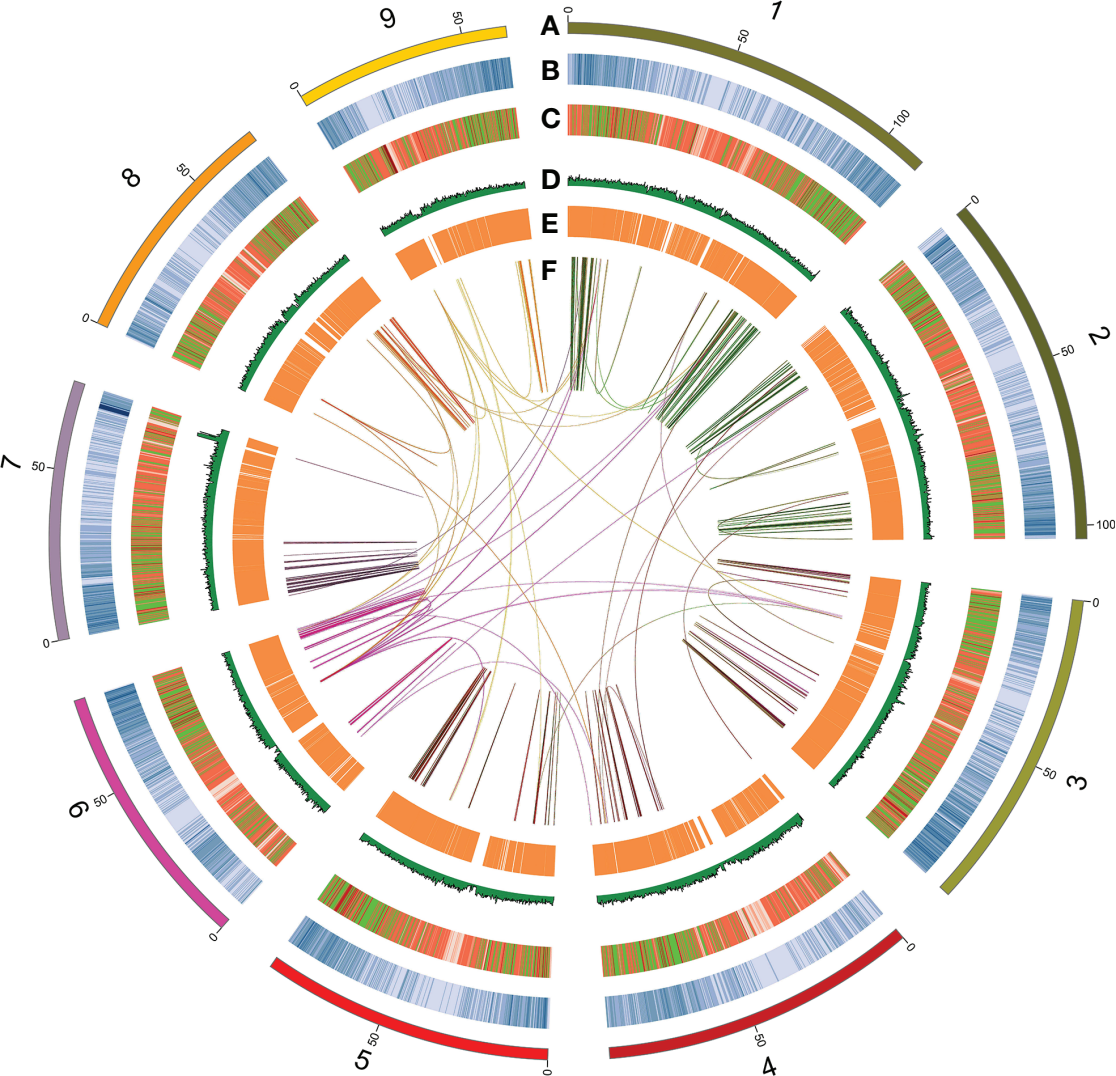
Circos plot showing the distribution of genomic features along the IMA1 genome. The rings from outermost to innermost indicate **(A)** nine pseudo-chromosomes of Beta vulgaris IMA1genome; **(B)** gene density distributed inside 200-kb sliding windows; **(C)** transposable element abundance; **(D)** distribution of GC content; **(E)** expression values of leaf-expressed genes; and **(F)** schematic presentation of major inter-chromosomal relations in the *B vulgaris* IMA1 genome. Each line represents a syntenic block; block size = 3 kb. Chromosomes in the outer ring are ordered by chromosomes length as follow:1, chr5; 2, chr4; 3, chr3; 4, chr7; 5, chr6; 6,chr9; 7, chr1; 8, chr8; 9, chr2.

**Figure 2:**
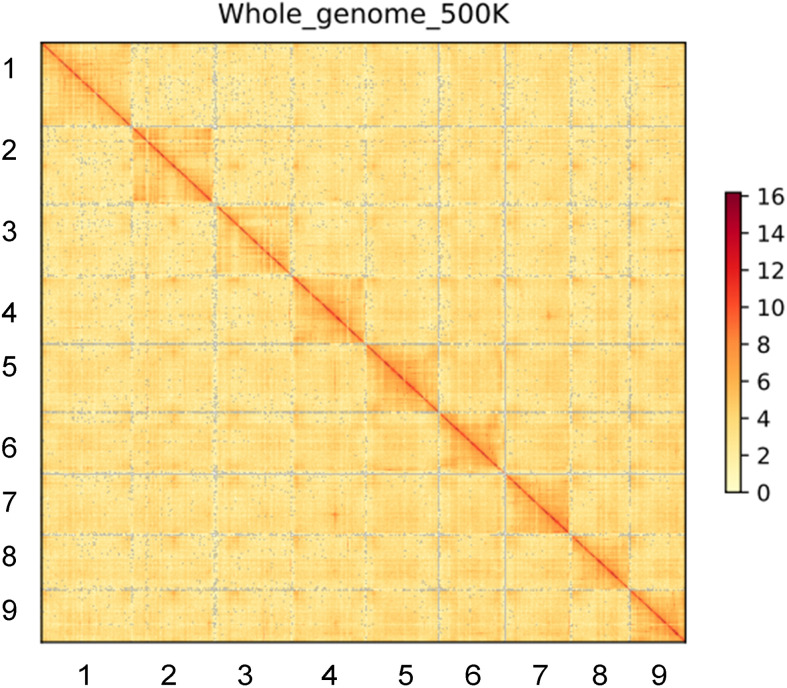
Integrated Hi-C interaction heatmap of *B. Vulgaris* IMA1 genome. The heatmap displays high-resolution single pseudo-chromosomes, which were scaffolded and composed independently. Lines are ordered by chromosomes length as follow:1, chr5; 2, chr4; 3, chr3; 4, chr7; 5, chr6; 6,chr9; 7, chr1; 8, chr8; 9, chr2.

The Illumina resequencing reads combined length was 67.22 Gb, which was 96× the estimated genome size. The RNA-seq generated a clean dataset of 15.93 Gb consisting of over 98.9 million Paired-end reads. Quality of Illumina resequencing reads was high (92.06% with Phred quality score >30). In total, 448 million high-quality, 150-bp clean paired-end reads were retained for use in the following analysis (**Supplementary Table 2**). The 17-mer analysis-based genome size of sugar beet was estimated at 720.5 Mb. A single main peak indicated the nature of the isolated genomic material, with heterozygosity of only 0.6% (**Supplementary Figure 1**). For accurate homozygous assembly, Illumina, Pacbio, and Hi-C sequences were combined to perform the sequencing. Approximately 120.75 Gb of clean data consisting of 805 million PE reads were produced from the Hi-C library sequencing (**Supplementary Table 2**). An initial 786-Mb genome sequence was obtained consisting of 4,824 contigs, with contig N50 of 367.5 kb. The longest contig was 5.91 Mb (**Table 1**). Additionally, 4,576 contigs from the Canu assembly were successfully clustered, ordered, and oriented to nine pseudo-chromosomes. In the IMA1 genome, 171 syntenic blocks were detected, which involved 3,508 genes (**Figures 1**, **2**). The results indicated the quality of the genome assembly for IMA1was high. The interaction signals were enriched in chromosomes, and the intensity of interaction along the diagonal was relatively smooth, showing well-organized contig orderings. The anchor rate was 99.1%, and only 248 contigs (7.1 Mb) were not anchored. The scaffold N50 was 93.06 Mb, and the longest chromosome values reached 112.63 Mb (**Supplementary Table 3**).

**Table 1:** Assembly statistics of *B. vulgaris* IMA1nuclear genome.

	Canu	HiC
Assembly genome size (Mb)	786.13	786.59
Genomic G+C content	35.85%	35.85%
Number of assembled scaffolds	4,824	257
Number of scaffolds (> 2 kb)	4,824	257
Max Length (Mb)	5.91	112.64
Scaffolds N50 (kb)	367.5	93.06

### Evaluation of the genome assembly

Assembled genomes were further validated by mapping NGS short reads, which indicated that 446.7 million (99.23%) Illumina reads were reliably aligned, which covered 96.84% of the assembly (**Supplementary Table 4**). Additionally, 96.8% to 98.08% of RNA-seq clean reads were reliably aligned to the assembled genome. Genome completeness was assessed based on the viridiplantae_odb9 database in the BUSCO program (Jia et al., 1997). A total of 1,326 (96.4%) complete single-copy orthologs among 1,375 conserved plant genes were recalled in the assembly (**Supplementary Table 5**). We assessed the coherence of the IMA1 genome assembly with LAI (Long terminal repeat assembly index). LAI score was assessed by LTR_RETRIEVER (v2.9.0) (Ou and Jiang, 2017). The LAI value of the IMA1 genome was 13.4, which was at the Reference level.

### Gene prediction and functional annotation

In the IMA1 genome, 35,003 genes encoding proteins were annotated. Average gene length was 1,121 bp. Total combined length of all genes was 39.23 Mb, which accounted for 4.99% of the assembled genome. According to the BUSCO assessment, 86.2% of core eukaryotic genes were complete in the assembly. Totals of 32,043; 27,574; 20,155; 10,157; and 21,351 genes were annotated in Nr, GO, COG, KEGG, and Swiss-Prot databases, respectively, and 32,047 (91.56%) genes had at least one hit to the databases (**Supplementary Table 6**). There were 8,725 genes annotated in all five databases, representing 24.93% of all protein-coding genes. Based on KEGG annotation (**Supplementary Figure 2**), 10,157 genes were involved in 33 pathways. There were 1,442 tRNAs, 945 5S rRNAs, 138 18S rRNAs, 139 28S rRNAs, 410 snRNAs, and 56 miRNAs in the IMA1 genome (**Supplementary Table 7**). The IMA1 genome contained a total of 512.72 Mb of repetitive sequences, with more than 284,501 tandem repeats identified (**Supplementary Table 8**).

### Comparisons of the IMA1 genome assembly with previously reported sugar beet genome assembly

The new sugar beet IMA1 assembled genome (~786 Mb) was compared with the two previously released chromosome-level assemblies of *B. vulgaris*: line RefBeet (~566 Mb, accession numbers: GCA_000511025.2) (Dohm et al., 2014) and EL10 (~540 Mb, accession numbers: GCA_002917755.1) (Funk et al., 2018). The new genome was much larger than those previously reported. In addition, the IMA1 genome had the longest chromosome length of 112.63Mb and the largest number of genes identified, with 35,003 genes. The two previous genome assemblies of *B. vulgaris* had scaffold N50 of 57.94 Mb and 2.01 Mb, respectively, which were much shorter than the 93.06 Mb in the current assembly (**Supplementary Table 3**). There were 257 scaffolds in the new genome assembly, and longer scaffold N50s were obtained than those in the EL10 and RefBeet genome, which was the best assembled genome to date. The completeness and continuity of the new assembly might be attributed to the high-sequencing depth of PacBio and Hi-C reads and the extremely low heterozygosity of the sugar beet line.

The IMA1 genome contained a total of 512.72 Mb of repetitive sequences, which were 65.18% of the IMA1 genome. It was higher than the previously released genome of sugar beet line EL10 and RefBeet (62.91% and 51.75%, respectively) (Dohm et al.,2014; Funk et al., 2018). The most abundant repetitive sequences in the IMA1 genome are Class I retroelement (66.65% of total TEs and 43.44% of genome). The Long terminal repeat retrotransposons (LTR-RTs) of IMA1 accounted for 31.24% of the assembly, while those of EL10 and RefBeet accounted for 28.07% and 21.82%, respectively. Over 284,501 tandem repeats were identified, representing 10.34% of the genome. (**Supplementary Table 8**). Compared with RefBeet and EL10, IMA1 annotated the highest proportion and number of repetitive sequences with significant improvements in the continuity and integrity of repeat regions.

**Figure 3:**
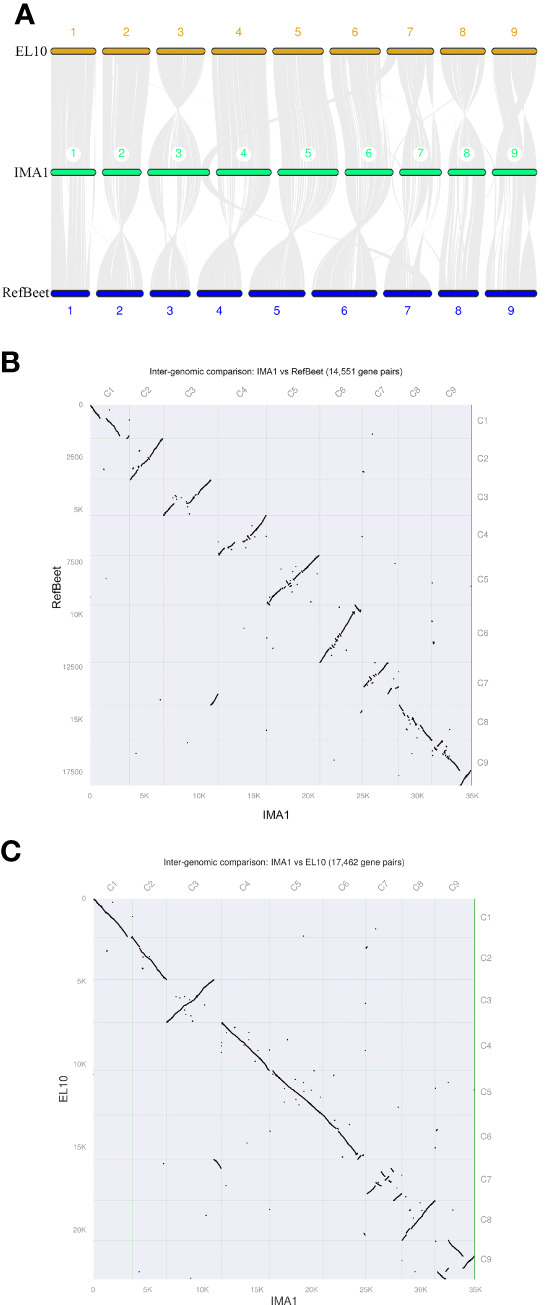
Genomic alignment among three genome assemblies of sugar beet lines IMA1, EL10, and RefBeet. **(A)** Schematic representation of synteny among IMA1, EL10, and RefBeet genomes. Gray lines connect matching gene pairs. **(B)** Scatter plot of syntenic blocks of conserved genes between *Beta vulgaris* IMA1 and RefBeet genomes. **(C)** Scatter plot of syntenic blocks of conserved genes between *B. vulgaris* IMA1 and EL10 genomes. Chromosome order in the new assembly was determined by length (from largest to smallest). Rightward and downward are 5′ to 3′ on assembly plus strands.

The synteny analysis showed that the *B. vulgaris* IMA1 assembly shared 17,462 and 14,551 common gene pairs with EL10 and RefBeet, respectively, indicating a high ratio of the syntenicregion. Most sequences in RefBeet and EL10 genomes aligned with corresponding counterparts in the IMA1 assembled genome; whereas the IMA1 assembly had extended sequences, especially in Chr1, Chr3, Chr4, and Chr7. Some genomic arrangements were also observed in the IMA1genome compared with RefBeet and EL10 (**Figure 3**).

### Evolution and gene family analysis of the *Beta vulgaris* IMA1 genome

**Figure 4:**
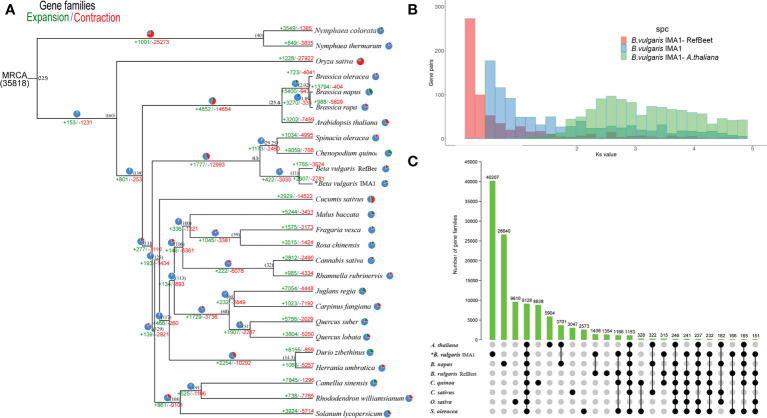
**(A)** Phylogenetic tree of gene families number unveiling expansion (green) and contraction (red) among 26 species. Pie diagrams represent the ratio of expanded (green), contracted (red), and conserved (blue) genes among whole gene families. The estimated divergence time (million years ago) is shown in black next to the phylogenetic tree. MRCA: most recent common ancestor. **(B)** Ks distributions for duplicated gene pairs in Beta vulgaris IMA1, RefBeet, and Arabidopsis thaliana. **(C)** UpSet plot of gene families intersection in *B. vulgaris* IMA1, RefBeet, *A. thaliana*, Brassica napus, Chenopodiumquinoa, Cannabis sativa, Oryza sativa, and Spinacia oleracea. Gene family numbers (clusters) are marked for each species and species intersection.

To analyze genome evolution and divergence time of IMA, some genome sequences of plant species were selected. Gene family expansions were greater than contractions in *Nymphaea colorata*, *Brassica napus*, *Chenopodium quinoa*, *B. vulgaris* IMA1, *Malusbaccata*, *Rosa chinensi*s, *Cannabis sativa*, *Juglansregia*, *Quercussuber*, *Duriozibethinus*, and *Camellia sinensis*, compared with the other species. In the phylogenetic tree, published *B. vulgaris* and IMA1 phylogenetically diverged into the Betoideae branch approximately 11 million years ago (Mya). Results also showed that published *B. vulgaris* and IMA1 were sisters in coccolithophores, which is consistent with the findings of phylogenetic analysis (**Figure 4A**).

Age distribution of duplicated genes was determined, followed by using a mixture model implemented in the mixtools R package (Benaglia et al, 2009) to identify significant gene duplication peaks consistent with whole genome duplications (WGDs). The median replication peak for IMA1 was around 0.55, which was younger than the ortholog divergence of IMA1 and *A. thaliana* (Ks, ~2.56) (**Figure 4B**). The distribution of ks values indicated that only one recent WGD event occurred in the IMA1 genome, whereas an ancient WGD event occurred 29 Mya ago.

From the 26 species, orthologous protein groups were delineated, and 35,818 orthologous groups were obtained (**Figure 4A**). In the IMA1 genome, 2,907 gene families expanded and 2,781 contracted. The 2,907 expanded gene families were annotated in KEGG and GO databases. In the GO analysis, the expanded orthologous groups were associated with biological regulation, growth, reproductive process, and signaling. In the KEGG analysis, most of the expanded genes were enriched to the categories of cell growth and death, plant hormone signal transduction, and environmental adaptation. The 2,781 contracted gene families were associated with signal transduction and steroid biosynthesis, as well as metabolism of pyruvate, terpenoids, polyketides, or lipids. In KEGG and GO analysis, contracted genes were also involved in developmental process and regulation of biological process.

In the comparison of IMA1, RefBeet, *A. thaliana*, *B. napus*, *C. quinoa*, *C. sativa*, *O. sativa*, and *S. oleracea*, 9,128 gene families were shared among these species (**Figure 4C**). According to the GO analysis, functions of those genes were primarily in growth, reproductive process, stimulus response, developmental process, and immune system. According to the KEGG analysis, enriched pathways for the genes included phenylpropanoid biosynthesis, purine metabolism, pyrimidine metabolism, and arginine biosynthesis.

### Phylogenetic analysis of *SWEET, SUT, SPS* and *SUS* gene families

**Figure 5:**
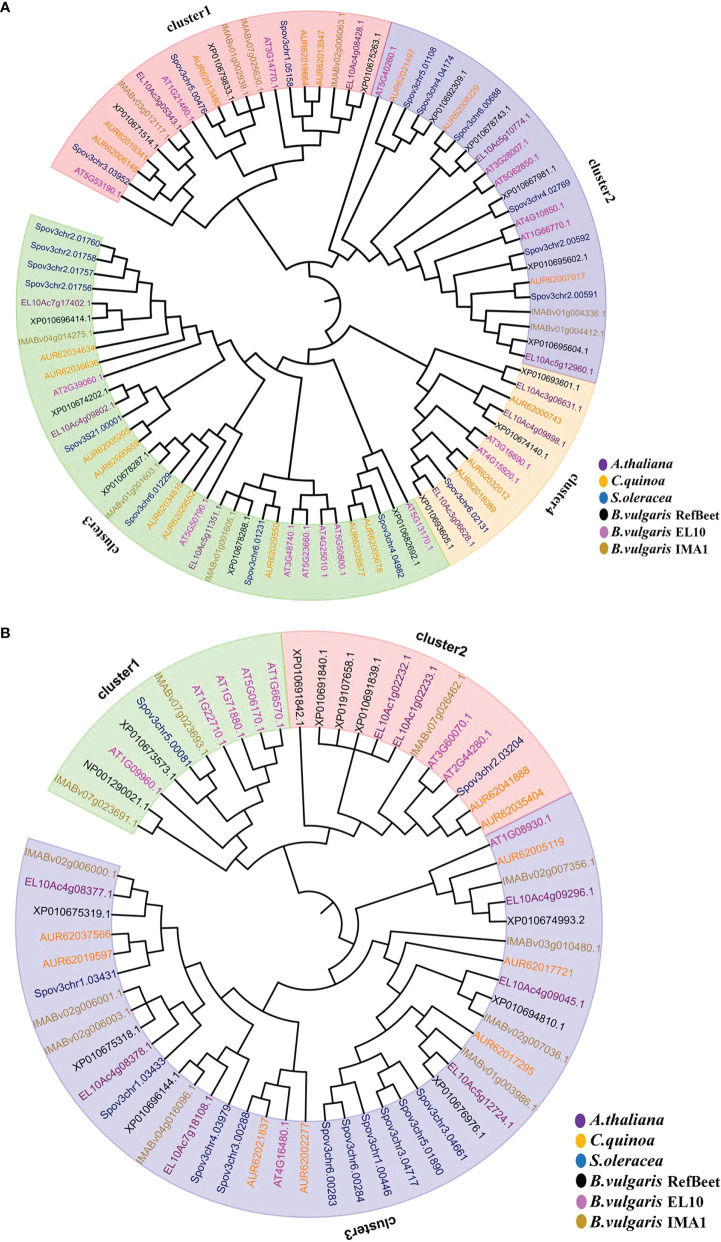
Genes in the *SWEET* and *SUT* families were clustered by neighbor-joining method. **(A)** Evolutionary tree of *SWEET* genes in *Arabidopsis thaliana*, *Chenopodium quinoa*, *Spinacia oleracea*, and *Beta vulgaris* IMA1, EL10, and RefBeet. **(B)** Evolutionary tree of *SUT* genes in *A. thaliana*, *C. quinoa*, *S.oleracea*, and *B. vulgaris* IMA1, EL10, and RefBeet.

To analyze evolutionary relations, a phylogenetic tree was constructed with *SWEET* (sugars will eventually be exported transporters) gene family members from *A. thaliana* (17), *B. vulgaris* IMA1 (9), RefBeet (16), and EL10 (10) (**Figure 5A**). Nine *SWEET* genes were found in the IMA1 genome, and they were grouped into four clusters: 1, 2, 3, and 4. In cluster 1,there were more subfamily genes of the *SWEET* family in IMA1 than in *B. vulgaris* RefBeet and EL10. Therefore, cluster 1 members from the *SWEET* family in IMA1 might have a more important role in sugar export transportation. In the *SUT* (sucrose transporters) gene family, a transmembrane transporter was involved in the absorption and transport of sucrose.

Evolutionary relations among *SUT* gene proteins from *B. vulgaris* IMA1(11), RefBeet(12), and EL10 (8) and *C. quinoa* (9), *S.oleracea* (12), and *A. thaliana* (9) were also determined *via* phylogenetic tree analysis (**Figure 5B**). The *SUT* gene proteins were classified into three groups, including clusters 1, 2, and 3 of subfamily genes. In cluster 3, *B. vulgaris* IMA1 had eight *SUT* genes, which was higher than that of RefBeet (6) and EL10 (6). It was hypothesized that the cluster 3 gene proteins have a key role in sucrose accumulation in IMA1.

Evolutionary relations among *SPS* (sucrose phosphate synthase) gene proteins from *B. vulgaris* IMA1(3), RefBeet (1), and EL10 (2) and *C. quinoa* (4),*Cucumissativus* (3), *B. napus* (5), *O. sativa* (4), *S.oleracea* (2), and *A. thaliana* (4) were determined *via* phylogenetic tree analysis (**Supplementary Figure 3**). The *SPS* gene proteins were divided into clusters 1, 2, and 3. In IMA1, clusters 3 and 2 had two and one *SPS* genes, respectively. In addition to *SWEET*, *SUT*, and *SPS* gene families, evolutionary relations of the *SUS* (sucrose synthase) gene family were also analyzed (**Supplementary Figure 4**). The *SUS* gene proteins were analyzed in *B. vulgaris* IMA1 (4), RefBeet (6), and EL10 (4) and *A. thaliana* (6), *C. quinoa* (7), and *S. oleracea* (4). Numbers in *SPS* and *SUS* gene families in IMA1 were fewer than those in other species. However, because IMA1 accumulated higher sugar content than that in other species, it was hypothesized that *SPS* and *SUS* gene family members in IMA1 had higher sugar accumulation efficiency than that in the other species.

### Genome wide association study of seven agronomic traits in *Beta vulgaris* IMA1

**Figure 6:**
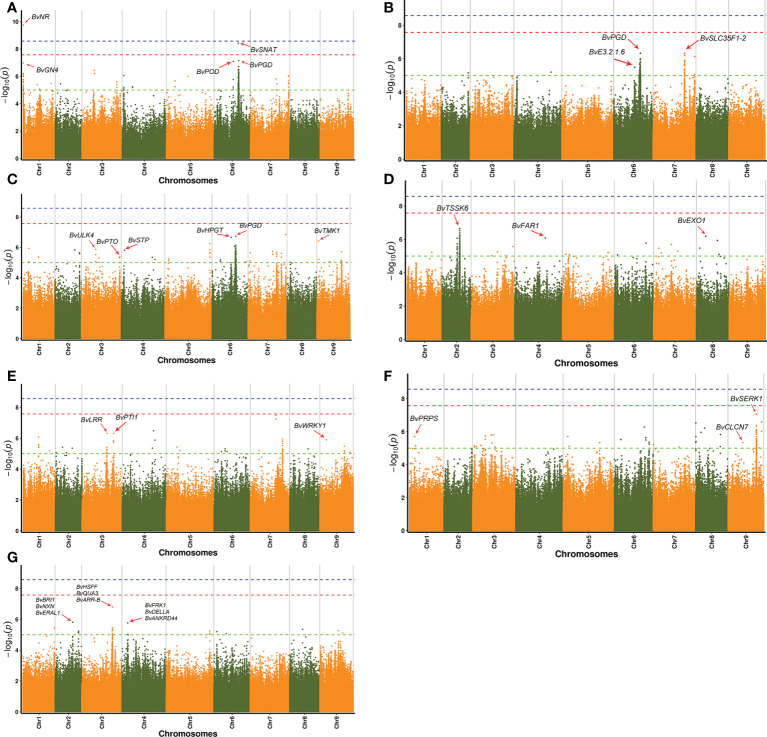
Manhattan plots for seven agronomic traits of 114 sugar beet lines. **(A)** Sugar content, **(B)** sugar yield per hectare, **(C)** root yield per hectare, **(D)** root rot of sugar beet, **(E)** damping off of sugar beet, **(F)** rhizomania of sugar beet, and **(G)** pollen fertility of sugar beet.

Phenotyping data of seven major agronomic traits of 114 *B. vulgaris* samples were used to perform GWAS (**Supplementary Table 9**). Sucrose content is an important economic trait for superior individuals of *B. vulgaris.* Nine strong GWAS signals were detected, including *BvNR* (IMABv01g023663), *BvGN4* (IMABv01g023668), *BvMYST1* (IMABv01g023671), *BvPGD* (IMABv01g018581), *BvSNAT* (IMABv01g018582), *BvCDK12_13* (IMABv01g018584), *BvGBF* (IMABv01g018599), *BvPOD*(IMABv01g018569), and *BvTOGT1*(IMABv01g018570) genes (**Figure 6A**; **Supplementary Figure 5A**; **Supplementary Table 10**).

Some strong GWAS signals on Chr6 and Chr7 were significantly associated with sugar yield per hectare, which is an important target in sugar beet breeding. For example, three genes were located in the strong association peaks, including *BvPGD* (IMABv01g018581), *BvE3.2.1.6* (IMABv01g018526), and *BvSLC35F1-2* (IMABv01g015264), which participate in the carbohydrate metabolism. The gene*BvYGK1* (IMABv01g018527) is associated with purine metabolism, and there was a strong GWAS signal on Chr7 for *BvACP7* (IMABv01g015268), which is associated with purple-acid phosphates (**Figure 6B**; **Supplementary Figure 5B**; **Supplementary Table11**). In addition, genes were also identified that were associated with root yield per hectare, including *BvPGD* (IMABv05g004241), *BvSTP* (IMABv04g007036), *BvHPGT* (IMABv05g004244), and glucose-6-phosphate 1-epimerase (IMABv05g004245), which were associated with the pentose phosphate pathway and galactose and monosaccharide transport. Serine/threonine protein kinase (STPK), a type of eukaryotic cell-like protein kinase, is involved in the transport of glucose and glutamine (Jia et al., 1997). Genes *BvULK4* (*IMABv03g010205*), *BvTMK1* (IMABv09g022186), and *BvPTO* (IMABv03g013119) code serine/threonine kinases and were also associated with root yield per hectare (**Figure 6C**; **Supplementary Figure 5C**; **Supplementary Table 12**).

Root rot, damping off, and rhizomaniaare emerging serious threats to sugar beet production. In the GWAS, several genes associated with disease defense were identified, including *BvTSSK6* (IMABv02g031103), *BvCLCN7* (IMABv09g022351), *BvPRPS* (IMABv01g024513), *BvEXO1* (IMABv08g027895), *BvFAR1* (IMABv04g006805), *BvSERK1* (IMABv09g023194), *BvLRR* (IMABv03g010906), *BvPTI1* (IMABv03g010905), *WRKY1* (IMABv09g020695), and *BvDELLA* (IMABv09g020694) (**Figures 6D–F**; **Supplementary Figures 5D–F**; **Supplementary Tables 13–15**).

In the GWAS analysis on pollen scale types of different beet varieties, there were some strong signals on Chr2, Chr3, and Chr4. Genes were identified that were related to pollen number, including *BvHSFF* (IMABv03g011676), *BvQUA3* (IMABv03g011680), *BvARR-B* (IMABv03g011683), *BvBRI1* (IMABv02g031269), *BvNXN* (IMABv02g031270), *BvERAL1*(IMABv02g031271), *BvFRK1*(IMABv04g005357), *BvDELLA*(IMABv04g005358), and *BvANKRD44* (IMABv04g005362). The gene *BvQUA3*, a putative homo-galacturonan methyl-transferase, is involved in regulating cell wall biosynthesis in *Arabidopsis* suspension-cultured cells (Miao et al., 2011). The gene *BvARR-B* is a member of the two-component response regulator ARR-B family, which is a partially redundant negative regulator of cytokinin signaling (To et al., 2004; Mason et al., 2005). The gene *BvBRI1*, protein brassinosteroid insensitive 1, is another gene associated with plant hormone signal transduction, which can transfer phosphorus-containing groups (Zipfel, 2008). The gene *BvFRK1*, a target of AtWRKY6 regulation during plant senescence, is a senescence-induced receptor-like serine/threonine-protein kinase (Robatzek and Somssich, 2002) (**Figure 6G**; **Supplementary Figure 5G**; **Supplementary Table 16**). The results provide valuable information on the characteristic genes associated with *B. vulgaris* pollen fertility, which can be used in molecular breeding.

### Gene ontology and KyotoEncyclopedia of genes and genomes pathway analysis of differential expressed genes

Transcriptomes of two pairs of sugarbeet cytoplasmic male sterility (CMS) lines were compared (MS137 vs. OT152 and MS301 vs. OT302). MS137 and MS301 are sugar beet sterile lines, and OT152 and OT302 are sugar beet maintainer lines. There were 2,032 and 2,090 significant DEGs identified in MS137 vs. OT152 and MS301 vs. OT302 comparisons, respectively (**Supplementary Figure 6**; **Supplementary Tables 17, 18**). Six hundred and twenty-one DEGs were identified in both MS137 vs. OT152 and MS301 vs. OT302 comparisons (**Supplementary Tables 19, 20**). In the KEGG analysis, the 621 shared genes were enriched in plant–pathogen interaction [two up-regulated genes, including *FRK1* (IMABv09g022061) and *RPS2* (IMABv03g008950)], glycolysis/gluconeogenesis [two down-regulated genes, including *pdhC* (newGene_5384) and *gapN* (IMABv04g008381)], photosynthesis [one down-regulated gene, *petF* (IMABv02g032492)), and MAPK signaling pathway (one up-regulated gene, *FRK1* (IMABv09g022061)]. “Binding” (GO:0005488, four up- and one down-regulated genes) and “catalytic activity” (GO:0003824, five up- and two down-regulated genes) were the two most enriched GO terms in the molecular function ontology. “Cell” (GO:0005623, two up- and one down-regulated genes) and “membrane part” (GO:0044425, three up- and two down-regulated genes) were the two most enriched GO termsin the cellular component. In addition, there were 997 up-regulated and 1,035 down-regulated DEGs in MS137 vs. OT152 (**Supplementary Table 17**) compared with 997 up-regulated and 1,093 DEGs in MS301 vs. OT302 (**Supplementary Table 18**). Among those DEGs, 334 were up-regulated and 285 were down-regulated between MS137 vs. OT152 and MS301 vs. OT302 (**Supplementary Figure 7**).

Based on GO and KEGG analyses of differential expression, six genes with significant differential expression were selected for a real-time fluorescence quantitative PCR test for verification (**Supplementary Figure 8**; **Supplementary Table 21**). The SGNH hydrolase gene (IMABv04g006046), GDSL esterase gene (IMABv05g001851), galacturonase gene (IMABv04g007649), and pectinlyase gene (IMABv07g016298) were up-regulated in maintainers. Genes for UDP-glucosyltransferase (IMABv06g016651) and cytochrome P450 (IMABv09g022885) were up-regulated in sterile lines. The results showed that expression profiles of the genes were consistent with transcriptome results.

## Discussion

The newly assembled genome was compared with the two previously released chromosome-level assemblies of *B. vulgaris*: line RefBeet (Dohm et al., 2014) and EL10 (Funk et al., 2018) (accession numbers: GCA_000511025.2 and GCA_002917755.1, respectively). The covered genome size of ~786 Mb was very close to the estimated sugar beet genome of 714 to 758 Mb (Arumuganathan and Earle, 1991) and was much larger than that of previous reports (RefBeet about ~540 Mb and EL10 about ~566 Mb). Compared with EL10, the best previously assembled genome, the new genome contained fewer scaffolds (257) and had a longer scaffold N50 (93.06Mb), indicating a significant improvement in sequence continuity.

When sugar beet IMA1 assembly and RefBeet genome were compared, the synteny analysis revealed that part of segments in Chr6 of IMA1 had inverted compared with the counterpart in Chr9 of RefBeet. Inherited variation between the two sugar beet cultivars and the much more accurate and complete assembly of IMA1 genome might be major reasons for differences. Overall, the quality of the new genome assembly of *B. vulgaris* IMA1was higher than that of the RefBeet genome, and therefore, it will be valuable in genetic analyses of sugar beet and related species.

The *SUS* and *SPS* gene families are well documented in plants, and gene family members vary from species to species (Castleden et al., 2004). In the metabolism of uridine diphosphate glucose, it is catalyzed and hydrolyzed to sucrose, and SPS is the key rate-limiting enzyme in the process (Lunn and Macrae, 2003). Changes in sugar content are closely related to expression levels of *SUS* and *SPS* genes (Lv et al., 2018). For example, increases inactivities of SUS and SPS enzymes are correlated with increases in sucrose content in the high sucrose-accumulating Japanese pear ‘Chojuro’. By contrast, activity of the enzymes does not increase in the low sucrose-accumulating pear cultivar ‘Yali’ during fruit ripening (Moriguchi et al., 1992). In addition, in the early stages of fruit development, Asian pear cultivars ‘Niitaka’ and ‘Whangkeumbae’ have relatively low sucrose content with relatively low activities of SUS and SPS enzymes, but when sucrose content reaches the peak value, SUS and SPS enzymes have the highest activities (Choi et al., 2009).

Cluster analysis of the *SUS* gene family in six dicotyledons was performed, and 31 genes were categorized into three different clusters. Six and four *SUS* genes were identified in sugar beet RefBeet and sugar beet IMA1, respectively. Similarly, *SPS* gene families were compared in nine dicotyledons, and 28 genes were categorized into three different clusters. Three and two *SPS* genes were identified in IMA1 and EL10, respectively, which were categorized to clusters 2 and 3, respectively. In addition, the number of *SPS* genes was species-related, and sugar metabolism regulation was related to the activity of SPS enzymes but was not affected by the quantity of genes.

In the distribution and transport of sucrose from source to sink in plants, sucrose transporters (*SUTs*) are important genes (Chao et al., 2020). However, the molecular mechanisms of SUT function in the sugar metabolism pathway are not fully understood. Three different SUT clusters have been identified in the analysis of *SUT* gene family clusters in eight dicot species (Chen et al., 2010).

The *SUS, SPS*, and *SUT* gene families are involved in sucrose synthesis, transport, and accumulation. Although there are fewer *SUS*, *SPS*, and *SUT* genes, sugar beet accumulates much more sugar in storage tissues than that of other dicots. Therefore, it was hypothesized that compared with other species, members of those gene families in sugar beet have more important roles in sugar catalysis and sugar transport efficiency or some strong transcription regulatory factors regulate those functional genes. As a result, sugarbeet has strong capability to synthesize, transport, and accumulate sugar.

The *SWEET* gene family in plants is categorized into four different clusters. SWEETs in cluster1 are mainly responsible for glucose transport. For example, AtSWEET1 of *Arabidopsis* can mediate the absorption and transport of glucose (Chong et al., 2014; Tao et al., 2015). SWEETs in cluster 2 are mainly responsible for monosaccharide transport (Chong et al., 2014). Most of the SWEETs in cluster3 are associated with sucrose transportation (Kryvoruchko et al., 2016). In *Arabidopsis*, AtSWEET11 and AtSWEET12 are responsible for transporting intracellular sucrose to the apoplast and then moving it into the phloem for long-distance transport (Chen et al., 2012). In cluster 4, AtSWEET16 is associated with transport of glucose, fructose, and sucrose (Klemens et al., 2013). In IMA1, 11 *SWEET* family genes were identified, including four in cluster1, one in cluster2, four in cluster3, and two in cluster4. It was hypothesized that the *SWEET* family genes in IMA1 are involved in transporting sucrose, fructose, and glucose, as well as long-distance transport from mesophyll cells into the phloem.

In summary, the data collected from gene sequencing of IMA1 were used to identify the members of *SUS*, *SPS*, *SUT*, and *SWEET* gene families that are generally considered to be crucial genes involved in plant sugar metabolism. Genes related to disease resistance were also identified. Candidate genes were nominated with the potential to regulate sugar metabolism and improve sugar productivity. Genes were also nominated that were related to disease-resistance, which could be targets for genetic improvement.

In this study, GWAS was performed for a set of sugar beet agronomic traits. Ten disease-resistance genes significantly associated with root rot, damping off, and rhizomania were identified. Five genes were identified that had significant relations with sugar yield per hectare of sugar beet. Among those genes, *BvSLC35F1-2* is involved in carbohydrate metabolism, whereas gene *BvACP7* codes a purple-acid phosphatase in a family of binuclear metallohydrolases identified in plants, animals, and fungi (Flanagan et al., 2006). In addition, nine highly expressed genes associated with sugar beet pollen fertility were identified. Those genes were involved in regulating cell wall biosynthesis, plant hormone signal transduction, and plant senescence. Among six significant DEGs, SGNH hydrolase, GDSL esterase, and pectinlyase were associated with another development, pollen wall development, and pollen tube growth (Guan et al., 2008; Wang et al., 2018; An et al., 2019). Those genes were down-regulated in sugar beet sterile lines, which might be related to sugar beet pollen abortion and male sterility. Plant auxin metabolism involves cytochrome P450 (Feldmann, 2001), and excessive auxin content can lead to stunting and sterility of plants. Cytochrome P450 was significantly up-regulated in sugar beet sterile lines, which might be related to sugar beet fertility. Yuan long Wu (Wu et al., 2022) recently identified a galacturan 1, 4-alpha-galacturonidase [EC:3.2.1.67] gene that controls the formation of cotton pollen outer cell wall. They revealed the important role of galacturan 1, 4-alpha-galacturonidaseis to de-esterify homogalacturonan in the formation of the outer wall of cotton pollen. In this study, galacturan1, 4-alpha-galacturonidase gene expression was up-regulated in MS301 in the MS301 vs. OT302 DEG analysis (**Supplementary Table 22**). There were multiple copies of the gene, and expression of all copies was up-regulated (IMABv01g025187, IMABv01g025166, IMABv01g025186, IMABv01g025168). However, in the analysis of MS137 *vs.* OT152 DEGs, there was no difference in expression of a galacturan1, 4-alpha-galacturonidase gene, suggesting that the mechanism of male sterility might be diverse. The results suggested that secondary metabolism regulates the expression of male sterility genes. The results also provide a valuable resource to study male sterility related pathways in sugar beet.

## Data availability statement

The data presented in the study are deposited in the Genome Sequence Archive (GSA) in National Genomics Data Center, Beijing Institute of Genomics (China National Center for Bioinformation), Chinese Academy of Sciences, accession number CRA002683. The final chromosome-level genome sequence data reported in this paper have been deposited in the Genome Warehouse (GWH) in National Genomics Data Center under accession number GWHAMMD00000000 that is publicly accessible at https://bigd.big.ac.cn/gwh.

## Author contributions

XL: investigation, resources, funding acquisition, supervision, and writing - review and editing. WH: methodology, supervision, funding acquisition, supervision, and writing - review and editing. JF: software, visualization, data curation, and writing – original draft. YL: resources. HZ: investigation, resources, and verification. DC: software and formal analysis. XW: investigation. ZZ: investigation and verification. LW: resources and data curation. PH: resources. BZ: investigation. TX: software and visualization. WZ: investigation. JH: formal analysis. CB: methodology, supervision, project administration, and funding acquisition. All authors contributed to the article and approved the submitted version.

## Funding

This study was supported by the Inner Mongolia Autonomous Region “the open competition mechanism to select the best candidates” project entitled “Creation of Elite Beet Germplasm and Breeding of Varieties Suitable for Mechanized Operation” (2022JBGS0029). This work was also funded by China Agriculture Research System of MOF and MARA, (CARA-170104 & CARA-170501).

## Acknowledgments

We would like to thank the editor and reviewers for their helpful comments on the manuscript.

## Conflict of interest

The authors declare that the research was conducted in the absence of any commercial or financial relationships that could be construed as a potential conflict of interest.

## Publisher’s note

All claims expressed in this article are solely those of the authors and do not necessarily represent those of their affiliated organizations, or those of the publisher, the editors and the reviewers. Any product that may be evaluated in this article, or claim that may be made by its manufacturer, is not guaranteed or endorsed by the publisher.
