# Supplementary material for: Genomic and transcriptomic-based analysis of agronomic traits in sugar beet (Beta vulgaris L.) pure line IMA1
Source: Front Plant Sci. 2022 Oct 13;13:1028885. doi: 10.3389/fpls.2022.1028885 (PMC9608375; doi:10.3389/fpls.2022.1028885)
Supplement: Supplementary file 1 [file DataSheet_1.docx]

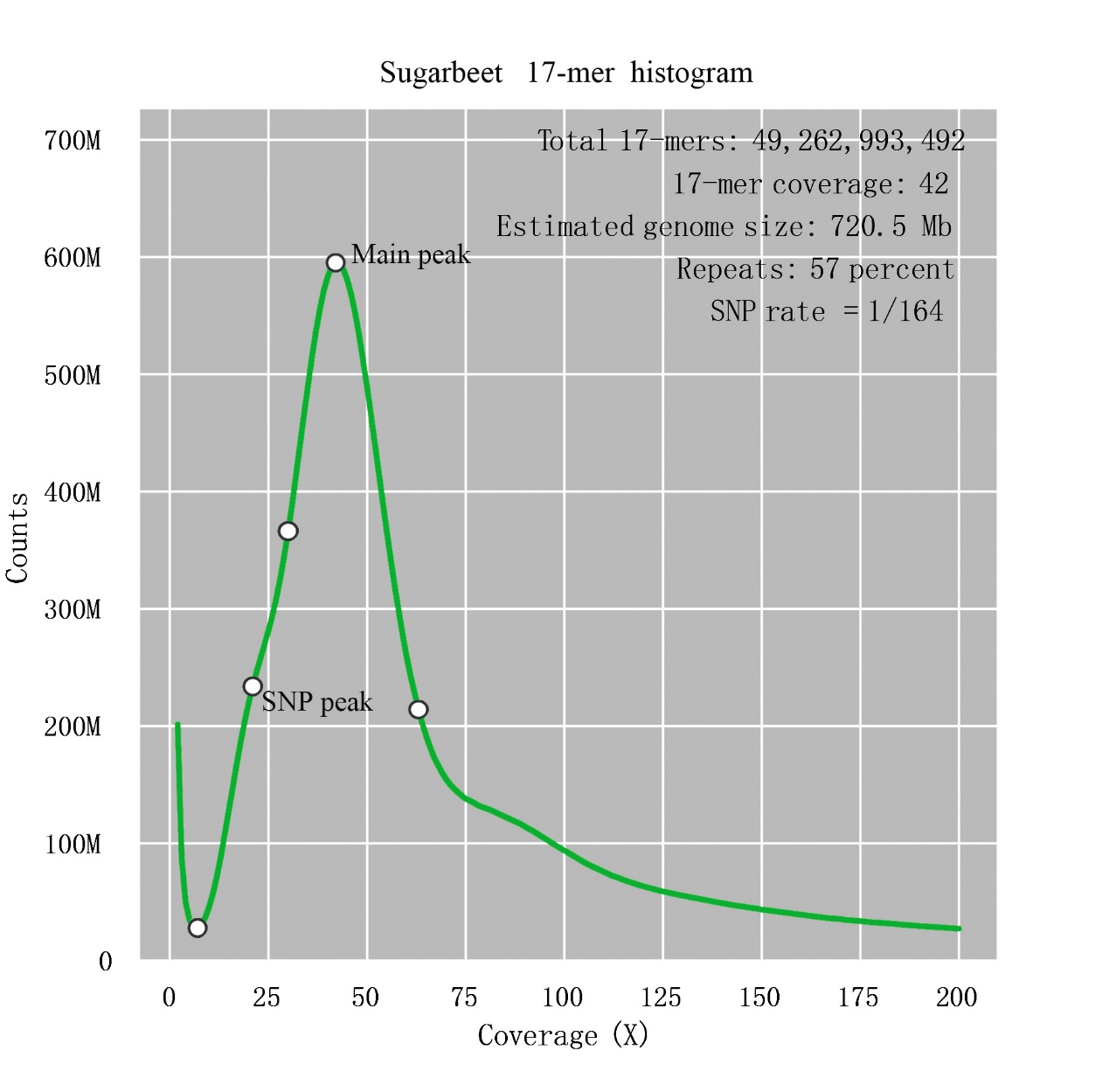


**Figure S1 K-mer frequency distribution curve.** All 17-mer sequences were extracted from pre-processed high-quality paired-end reads and plotted the frequency (y-axis) as a function of the depth (x-axis). The single main peak indicates a homozygous genomic source.


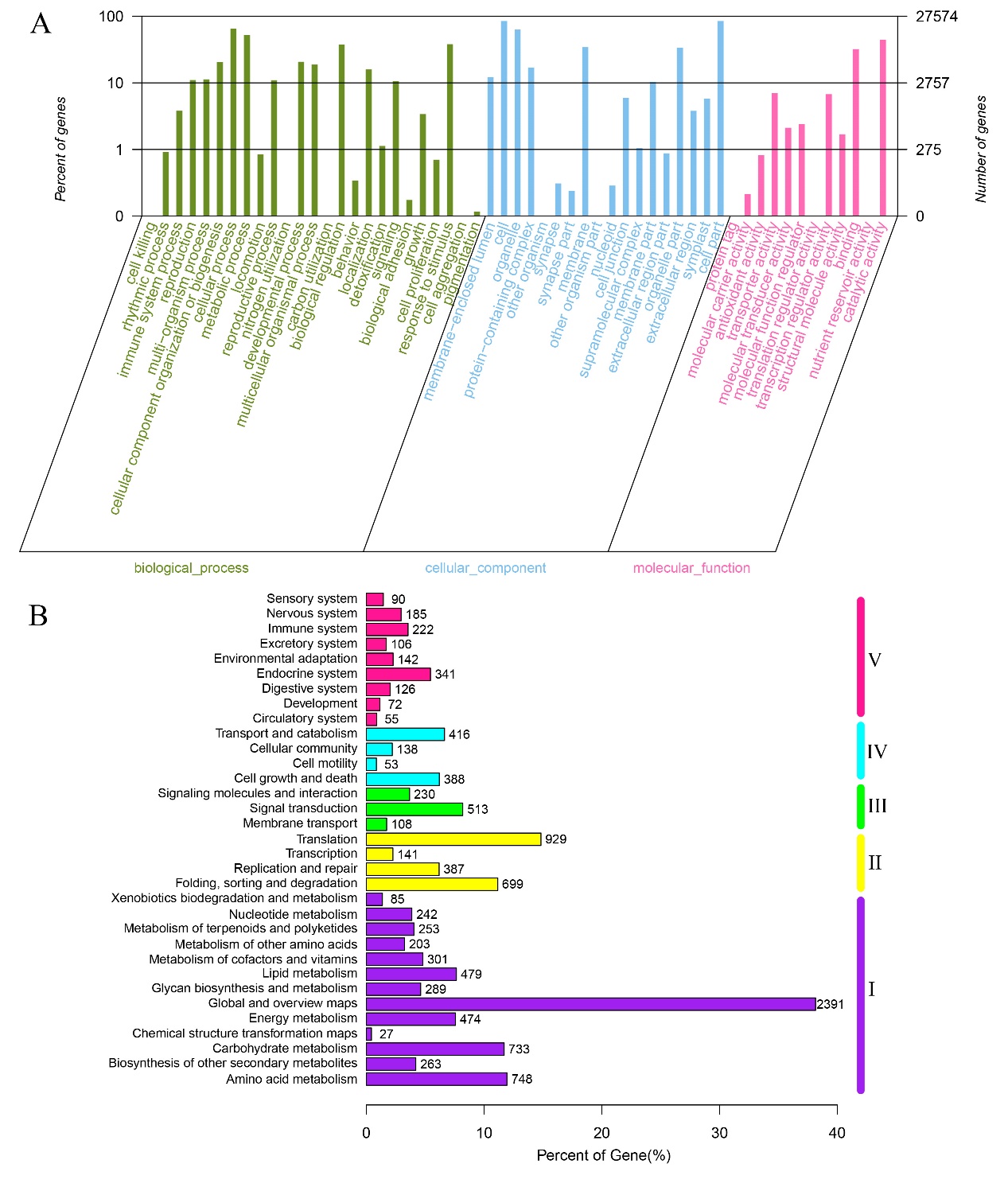


**Figure S2 Functional annotation of *B. vulgaris* IMA1 protein coding genes**. **A.** Level 2 GO annotation of protein-coding genes in *B. vulgaris* IMA1 genome. The assigned GO terms belonged to three major ontologies: molecular function (MF), biological process (BP) and cellular component (CC). **B.** KEGG pathway annotation of protein-coding genes in *B. vulgaris* genome. I: Metabolism; II: Genetic Information Processing; III: Environmental Information Processing; IV: Cellular Processes; V: Organismal Systems.


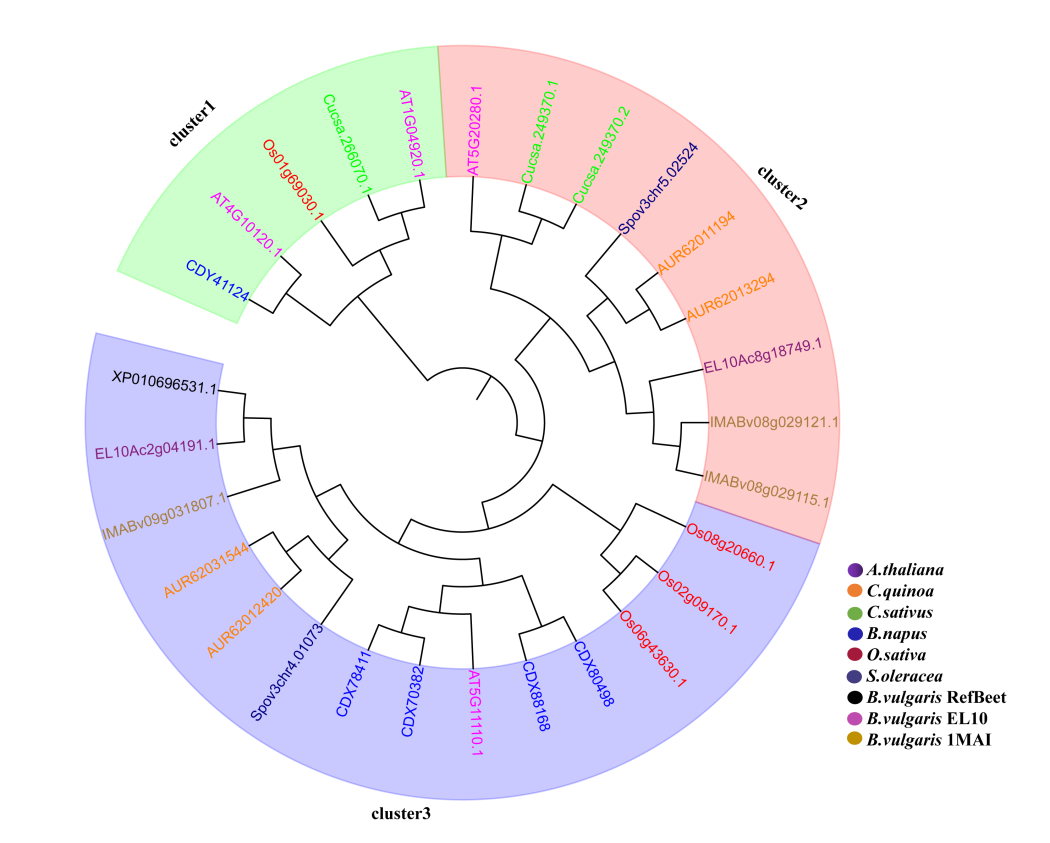


**Figure S3 Evolutionary tree of *SPS* genes in *A. thaliana, C. quinoa, C.sativus, B.napus, O.sativa, S. oleracea, B. vulgaris* IMA1, EL10 and RefBeet*.*** Genes in the *SPS* family were separately clustered using MEGA7 software via the neighbor-joining method.


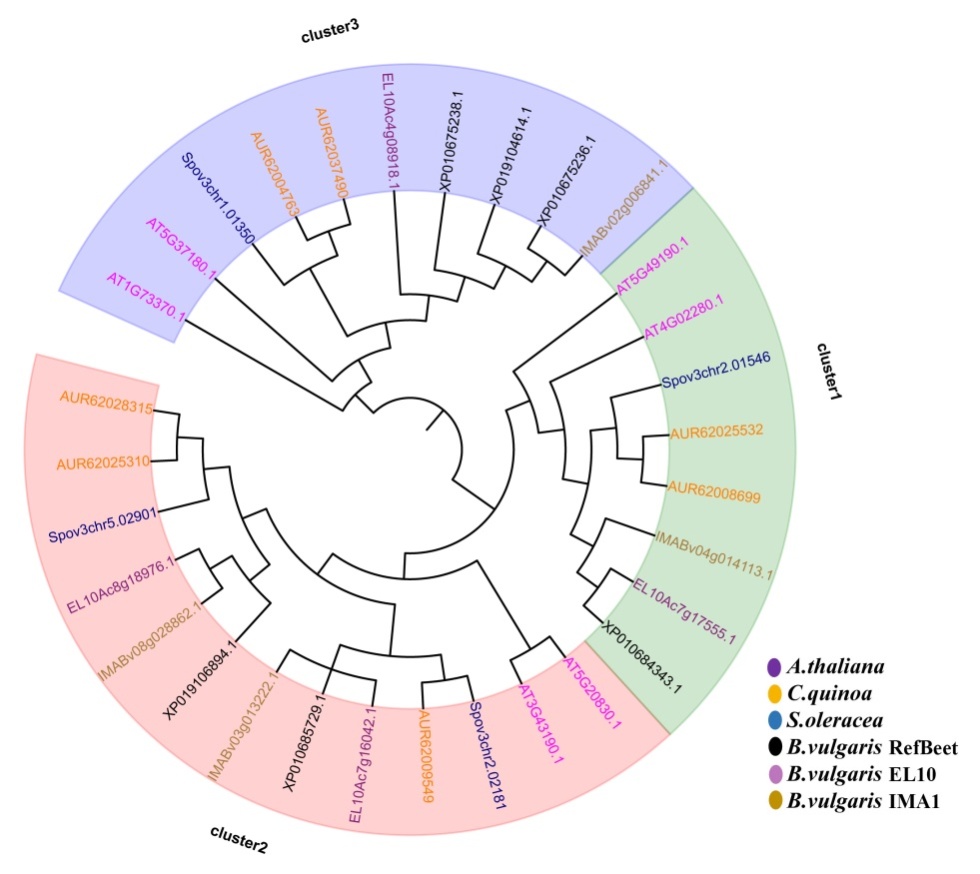


**Figure S4 Evolutionary tree of *SUS* genes in *A. thaliana, C. quinoa, Spinacia oleracea, B. vulgaris* IMA1, EL10 and RefBeet*.*** Genes in the *SUS* family were separately clustered using MEGA7 software via the neighbor-joining method.


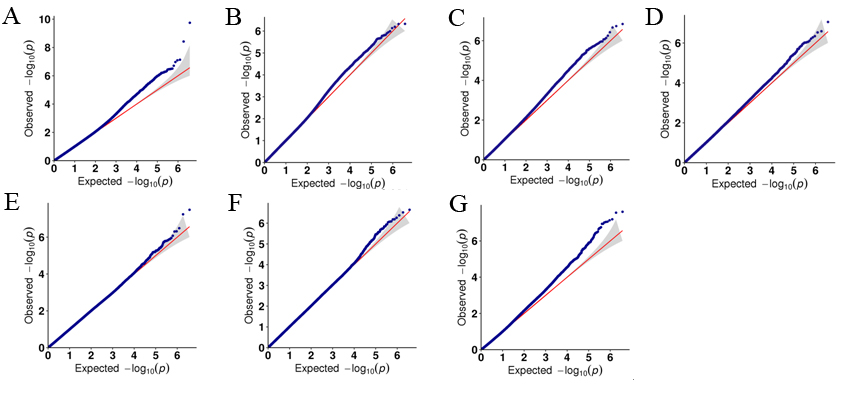


**Figure S5 Seven important agronomic traits qq plot** A. qq plot of sugar content; B. qq plot of sugar yield (kg/ha); C. qq plot of root yield (kg/ha); D. qq plot of rhizomania of sugar beet; E. qq plot of root rot of sugar beet; F. qq plot of damping off of sugar beet; G. qq plot of pollen fertility of sugar beet.


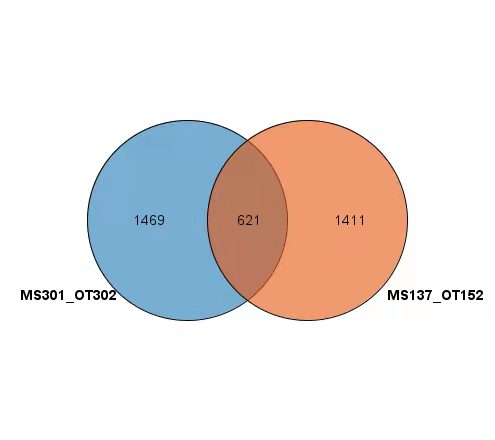


**Figure S6 Venn diagram showing the number of DEG of two pairs of sugar beet CMS lines**


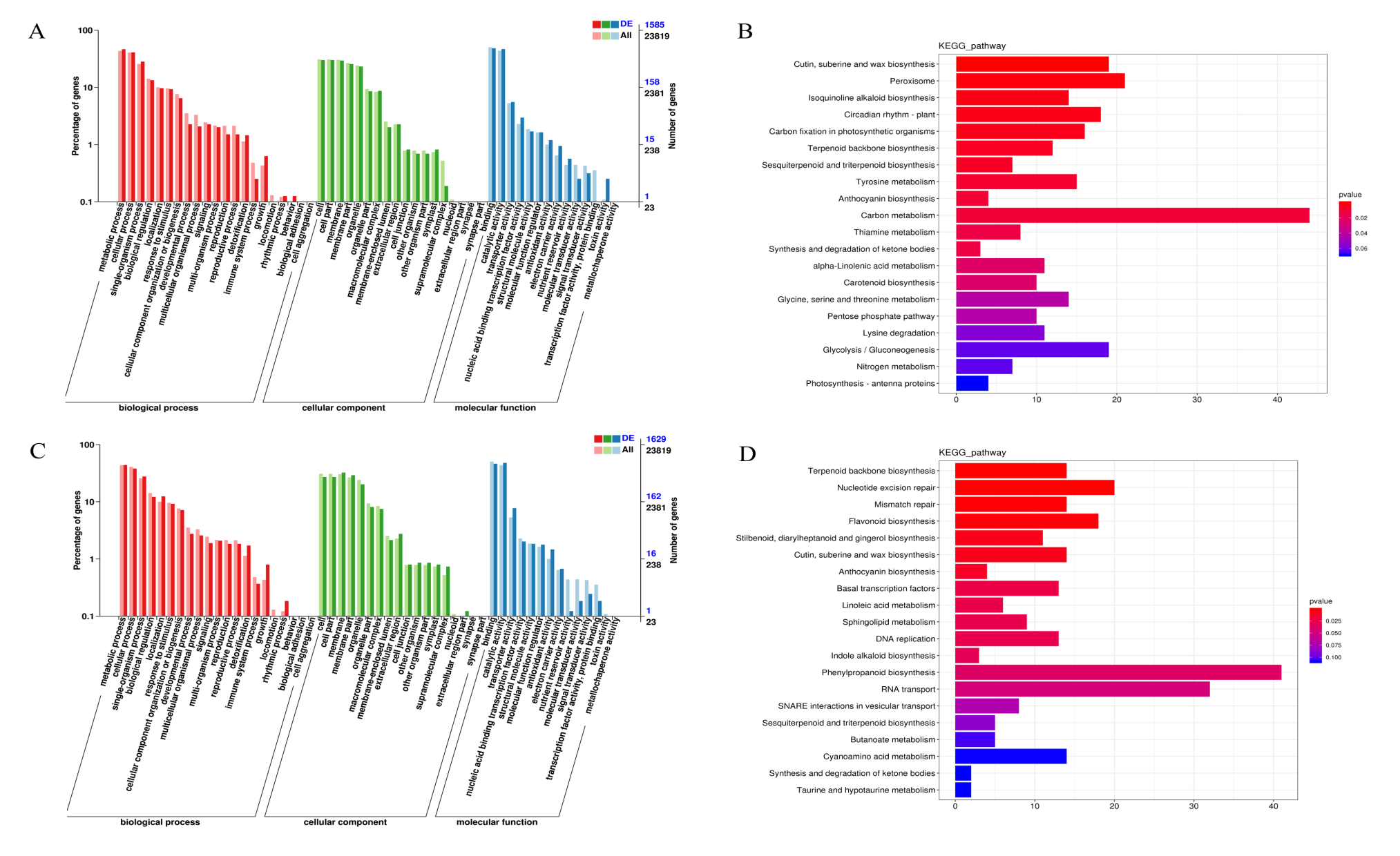


**Figure S7 GO terms and KOGG pathways enrichment analysis of two pairs of sugar beet CMS lines trancriptome (A. GO terms analysis of MS137 vs OT152; B. KOGG pathways analysis of MS137 vs OT152; C. GO terms analysis of MS301 vs OT302; D. KOGG pathways analysis of MS301 vs OT302.**


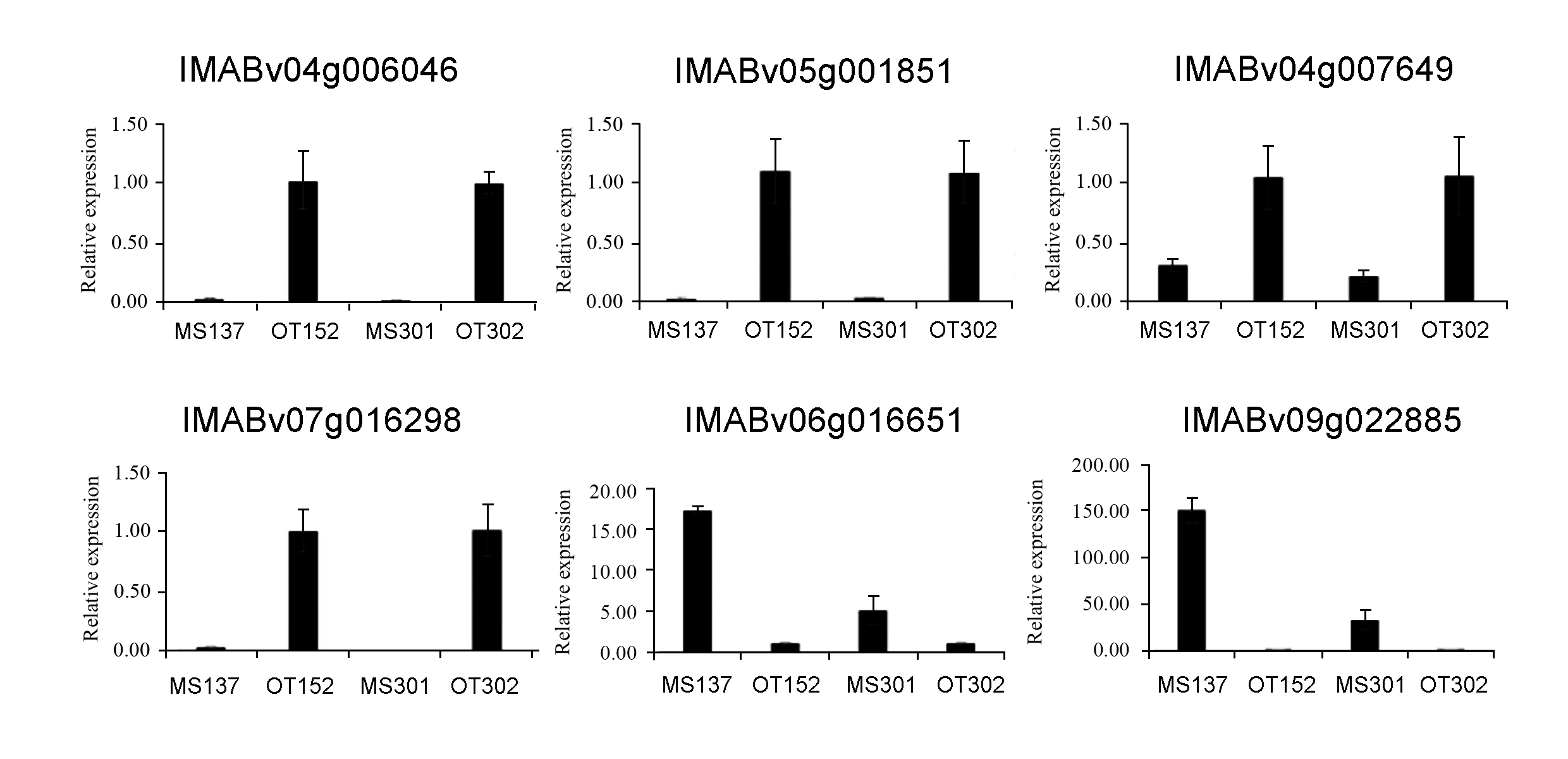


**Figure S8 q-PCR validation of 6 differentially expressed genes**
