# Supplementary material for: Genomic and transcriptomic-based analysis of agronomic traits in sugar beet (Beta vulgaris L.) pure line IMA1
Source: Front Plant Sci. 2022 Oct 13;13:1028885. doi: 10.3389/fpls.2022.1028885 (PMC9608375; doi:10.3389/fpls.2022.1028885)
Supplement: Supplementary file 2 [file Table_1.docx]

Sugar beet IMA1 Supplementary information

**Table S1. Statistics of PacBio sequel clean subreads data**

| **Items** | **Clean data** |
| --- | --- |
| Sequencing platform | PacBio SEQUEL |
| Total size (bp) | 65,669,711,775 |
| Subreads Number | 6,121,805 |
| Largest Length (bp) | 87,306 |
| N50 Length (bp) | 17,047 |
| N90 Length (bp) | 6,292 |
| Average Length (bp) | 10,727 |
| Mean GC (%) | 39 |
| Coverage (×) | 93.81 |

|  | **Genomic DNA-seq** | |  | **RNAseq1** | |  | **RNAseq2** | |  | **RNAseq3** | |  | **HiC DNA-seq** | |
| --- | --- | --- | --- | --- | --- | --- | --- | --- | --- | --- | --- | --- | --- | --- |
|  | **Raw data** | **Clean data** |  | **Raw data** | **Clean data** |  | **Raw data** | **Clean data** |  | **Raw data** | **Clean data** |  | **Raw data** | **Clean data** |
| Sequencing platform | IlluminaHiSeq X Ten (PE) | |  | IlluminaHiSeq X Ten (PE) | | | | | | | |  | IlluminaHiSeq X Ten (PE) | |
| Total size (Gbp) | 68.23 | 67.22 |  | 5.58 | 5.51 |  | 5.29 | 5.20 |  | 5.29 | 5.22 |  | 143.15 | 120.75 |
| Clean reads rates (%) |  | 98.51 |  |  | 98.66 |  |  | 98.36 |  |  | 98.73 |  |  | 84.35 |
| Total reads number | 454,878,824 | 448,100,352 |  | 37,200,074 | 36,700,324 |  | 35,260,214 | 34,683,308 |  | 35,239,546 | 34,793,434 |  | 954,317,194 | 804,969,910 |
| Reads length (bp) | 150 | 150 |  | 150 | 150 |  | 150 | 150 |  | 150 | 150 |  | 150 | 150 |
| Q30 (%) | 92.06 | 92.23 |  | 92.09 | 92.27 |  | 93.05 | 93.22 |  | 93.21 | 93.39 |  | 92.27 | 93.26 |
| Coverage (×) | 97.47 | 96.03 |  | - | - |  | - | - |  | - | - |  | - | - |

**Table S2. Statistics of resequencing, RNA-Seq and HiC DNA-Seq data on Illumina HiSeq X Ten platform**

**Table S 3. Comparison between genome assembly quality of *B. vulgaris* IMA1、EL10 and RefBeet**

| **Strategy** |  | **IMA1（PacBio+Bionano+Hi-C）** | | | | | |  |  | **EL10（PacBio+Bionano+Hi-C）** | | | |  |  | **RefBeet（Illumina）** | | | |  |
| --- | --- | --- | --- | --- | --- | --- | --- | --- | --- | --- | --- | --- | --- | --- | --- | --- | --- | --- | --- | --- |
| **Chromosome** | **Chromosome Length (Mb)** | | | **Contig Num.** | | **Gene Num.** | **N50 Size (Mb)** | | **Chromosome Length (Mb)** | | **Contig Num.** | **Gene Num.** | **N50 Size (Mb)** | | **Chromosome Length (Mb)** | | **Contig Num. *** | **Gene Num.** | **N50 Size (Mb)** | |
| **Chr1** | 112.63 | | 847 | | 4,887 | | 93.06 | | 58.09 | | 47 | 3,190 | 57.94 | | 51.60 | | **-** | 1,774 | 2.01 | |
| **Chr2** | 103.97 | | 824 | | 4,424 | |  |  | 54.97 | | 30 | 3,211 |  |  | 50.45 | | **-** | 2,192 |  |  |
| **Chr3** | 94.94 | | 643 | | 5,002 | |  |  | 54.10 | | 22 | 3,237 |  |  | 40.36 | | **-** | 1,808 |  |  |
| **Chr4** | 93.06 | | 698 | | 3,366 | |  |  | 61.16 | | 47 | 3,530 |  |  | 53.44 | | **-** | 2,066 |  |  |
| **Chr5** | 85.64 | | 395 | | 3,870 | |  |  | 59.22 | | 30 | 3,380 |  |  | 65.39 | | **-** | 2,672 |  |  |
| **Chr6** | 79.00 | | 405 | | 3,593 | |  |  | 65.10 | | 52 | 3,500 |  |  | 65.62 | | **-** | 3,021 |  |  |
| **Chr7** | 75.99 | | 322 | | 3,613 | |  |  | 57.35 | | 40 | 3,162 |  |  | 54.72 | | **-** | 2,225 |  |  |
| **Chr8** | 71.24 | | 226 | | 3,017 | |  |  | 57.94 | | 37 | 3,118 |  |  | 48.03 | | **-** | 1,896 |  |  |
| **Chr9** | 63.00 | | 216 | | 3,113 | |  |  | 52.18 | | 28 | 3,046 |  |  | 50.18 | | **-** | 2,404 |  |  |
| **Subtotal** | 779.48 | | 4,576 | | 34,885 | |  | | 520.11 | | 333 | 29,374 |  | | 479.79 | | **-** | 20,058 |  | |
| **Other contigs** | 7.10 | | 248 | | 118 | |  | | 20.42 | | 31 | 1,100 |  | | 86.39 | | **-** | 8,055 |  | |
| **Total** | 786.59 | | 4,824 | | 35,003 | |  | | 540.53 | | 364 | 30,474 |  | | 566.18 | | **-** | 28,113 |  | |

* RefBeet's Contigo Num. data wasn’t included in the literature.

**Table S4. Assessment of genome consistency by Illumina short reads**

| **Items** | **Statistics** |
| --- | --- |
| Total Reads | 450,153,349 |
| Total Raw Date (Mb) | 67,319.92 |
| Mapped Reads | 446,708,372 |
| Fraction of Mapped Reads (%) | 99.23 |
| Mapped bases (Mb) | 67,006.26 |
| Fraction of Mapped bases (%) | 99.53 |
| Genome Length (Mb) | 791.6 |
| Mean Depth | 84 |
| Coverage Rate (%) | 96.84 |

**Table S5. BUSCO analysis of *B. vulgaris* IMA1 Genome completeness**

| **Description** | **Number** | **Percentage (%)** |
| --- | --- | --- |
| Complete BUSCOs(C) | 1326 | 96.4 |
| Complete and single-copy BUSCOs(S) | 1278 | 92.9 |
| Complete and duplicated BUSCOs(D) | 48 | 3.5 |
| Fragmented BUSCOs(F) | 11 | 0.8 |
| Missing BUSCOs(M) | 38 | 2.8 |
| Total BUSCO groups searched | 1375 | 100 |

**Table S6. Annotation statistics for the *B. vulgaris* IMA1 genome**

| **Annotation statistics for the genome** | | **Number** | **Percentage** |
| --- | --- | --- | --- |
|  | Total protein-coding genes | 35,003 |  |
|  | Nr | 32,043 | 91.54% |
|  | GO | 27,574 | 78.78% |
|  | COG | 20,155 | 57.58% |
|  | KEGG | 10,157 | 29.02% |
|  | Swiss-Prot | 21,351 | 61.00% |
|  | In all databases | 8,725 | 24.93% |
|  | At least one database | 32,047 | 91.56% |

**Table S7. Statistical analysis of non-coding RNAs in *B. vulgaris* IMA1 genome**

| **ncRNA Items** | **Number** |
| --- | --- |
| tRNAs | 1,442 |
| snRNA | 410 |
| 5S rRNAs | 945 |
| 18S rRNAs | 138 |
| 28S rRNAs | 139 |
| miRNAs | 56 |

**Table S 8. Comparison of repetitive element annotations of *B. vulgaris* IMA1, EL10 and RefBeet**

|  |  | **IMA1** | | | |  |  | **EL10** | | | |  |  | **RefBeet** | | | |  |
| --- | --- | --- | --- | --- | --- | --- | --- | --- | --- | --- | --- | --- | --- | --- | --- | --- | --- | --- |
|  | **No. of TEs** | | **Length (Mb)** | **% of total TEs** | **% of genome** | | **No. of TEs** | | **Length (Mb)** | **% of total TEs** | **% of genome** | | **No. of TEs** | | **Length (Mb)** | **% of total TEs** | **% of genome** | |
| Total repeat fraction | 1,228,472 | | 512.72 | 100 | **65.18** | | 844,181 | | 340.38 | 100 | **62.91** | | 865,265 | | 293.21 | 100 | **51.75** | |
| **Class I: Retroelement** | 551,567 | | 341.72 | 66.65 | 43.44 | | 413,488 | | 230.00 | 67.57 | 42.51 | | 443,909 | | 203.54 | 69.42 | 35.93 | |
| **LTR Retrotransposon** | 289,321 | | 245.73 | 47.93 | 31.24 | | 184,550 | | 151.89 | 44.62 | 28.07 | | 201,298 | | 123.60 | 42.15 | 21.82 | |
| **non-LTR Retrotransposon** | 165,809 | | 72.12 | 14.07 | 9.17 | | 142,950 | | 57.01 | 16.75 | 10.54 | | 152,174 | | 58.42 | 19.92 | 10.31 | |
| **unclassified retroelement** | 96,437 | | 23.87 | 4.66 | 3.04 | | 85,988 | | 21.10 | 6.20 | 3.90 | | 90,437 | | 21.53 | 7.34 | 3.80 | |
| **Class II: DNA Transposon** | 352,936 | | 92.73 | 18.09 | 11.79 | | 209,463 | | 65.37 | 19.21 | 12.08 | | 21,9074 | | 63.04 | 21.50 | 11.13 | |
| **Helitron** | 7,001 | | 4.33 | 0.84 | 0.55 | | 6,258 | | 3.83 | 1.13 | 0.71 | | 2,525 | | 1.47 | 0.50 | 0.26 | |
| **Tandem Repeats** | 284,501 | | 81.37 | 15.87 | 10.34 | | 15,8791 | | 37.60 | 11.05 | 6.95 | | 14,6832 | | 20.21 | 6.89 | 3.57 | |
| **Unkown** | 33,719 | | 10.56 | 2.06 | 1.34 | | 33,350 | | 10.67 | 3.13 | 1.97 | | 34,046 | | 10.48 | 3.57 | 1.85 | |

**Table S9. Phenotypic data of 7 agronomic traits of 114 *B. vulgaris* samples**

**Table S10. Sugar content sign loci**

**Table S11. Sugar yield sign loci**

**Table S12. Root yield sign loci**

**Table S13. Root rot sign loci**

**Table S14. Damping off sign loci**

**Table S15. Rhizomania sign loci**

**Table S16. Pollen Fertility sign loci**

**Table S17. 2032 DEGs in MS137 VS OT152**

**Table S18. 2090 DEGs in MS301 VS OT302**

**Table S19. 621 DEGs identified in MS137 VS OT152**

**Table S20. 621 DEGs identified in MS301 VS OT302**

**Table S21. The analysis of DGEs**

**Table S22. Galacturonan 1,4-α-galacturonidase gene expression up-regulated in MS301 from MS301 VS OT302**
